# Fetal Programming and Sexual Dimorphism of Mitochondrial Protein Expression and Activity of Hearts of Prenatally Hypoxic Guinea Pig Offspring

**DOI:** 10.1155/2019/7210249

**Published:** 2019-06-02

**Authors:** Loren P. Thompson, Hong Song, Brian M. Polster

**Affiliations:** ^1^Department of Obstetrics, Gynecology and Reproductive Sciences, University of Maryland, Baltimore, School of Medicine, 655 W. Baltimore St., Baltimore, MD 21201, USA; ^2^Department of Anesthesiology and Center for Shock, Trauma and Anesthesiology Research, University of Maryland, Baltimore, School of Medicine, 655 W. Baltimore St., Baltimore, MD 21201, USA

## Abstract

Chronic intrauterine hypoxia is a programming stimulus of cardiovascular dysfunction. While the fetal heart adapts to the reduced oxygenation, the offspring heart becomes vulnerable to subsequent metabolic challenges as an adult. Cardiac mitochondria are key organelles responsible for an efficient energy supply but are subject to damage under hypoxic conditions. We propose that intrauterine hypoxia alters mitochondrial function as an underlying programming mechanism of contractile dysfunction in the offspring. Indices of mitochondrial function such as mitochondrial DNA content, Complex (C) I-V expression, and CI/CIV enzyme activity were measured in hearts of male and female offspring at 90 days old exposed to prenatal hypoxia (10.5% O_2_) for 14 d prior to term (65 d). Both left ventricular tissue and cardiomyocytes exhibited decreased mitochondrial DNA content, expression of CIV, and CI/CIV activity in male hearts. In female cardiomyocytes, hypoxia had no effect on protein expression of CI-CV nor on CI/CIV activity. This study suggests that chronic intrauterine hypoxia alters the intrinsic properties of select respiratory complexes as a programming mechanism of cardiac dysfunction in the offspring. Sex differences in mitochondrial function may underlie the increased vulnerability of age-matched males compared to females in cardiovascular disease and heart failure.

## 1. Introduction

In adult hearts, the mitochondria play an important role in contractile function in generating 90% of ATP via oxidative phosphorylation [1, 2]. Since the heart has a relatively low ATP content and a high energy demand [1], the generation and delivery of the energy supply to the myofibrils must be highly efficient. In contrast to the adult, the early fetal heart relies predominantly on glycolysis for its energy supply because (1) glucose is a major energy substrate, (2) the glycolytic enzymes are upregulated via hypoxia signaling [3], and (3) oxidative phosphorylation is inefficient, resulting from a less organized ultrastructure within the mitochondria and in association with the myofibrils [4, 5]. However, despite the reliance on glycolysis as a metabolic pathway, oxidative capacity of the fetal heart is still important as the heart undergoes a metabolic switch to oxidative phosphorylation in preparation to and following birth [3, 6–11]. Thus, intrauterine stressors that alter the normal fetal heart growth pattern and cellular organization may alter the maturational progress of fetal cardiac metabolism by disrupting both myofibrillar development and mitochondrial function.

The adult heart relies predominantly on the TCA cycle and *β*-oxidation to provide NADH and FADH_2_ to the electron transport chain for oxidative phosphorylation. Under normal conditions, electrons are transported from CI to CIV, where O_2_ is reduced to H_2_O, and a H+ gradient is generated by CI, CIII, and CIV, which is the driving force for ATP synthase activity [1]. The efficiency of ATP generation is dependent on the efficiency of electron flux along the respiratory chain and O_2_ reduction by cytochrome c oxidase within the inner mitochondrial membrane. Altered expression of mitochondrial protein and altered respiratory complex activity can result in reduced electron flux, excess electron accumulation, and generation of superoxide anions from CI, CIII, and TCA cycle dehydrogenase enzymes sensitive to the NADH/NAD+ ratio [12, 13]. These events lead to reduced ATP synthesis [1]. In both animal and human studies, altered cardiac mitochondrial protein expression and activity are associated with heart dysfunction and failure, contributing to cardiovascular disease [14–19].

Now, decades of research have established the negative impact of intrauterine stress on fetal growth and development and its lasting consequences of increased risk of adult cardiovascular disease [20, 21]. Early epidemiological studies have provided insight into cardiovascular programming, showing that intrauterine growth restriction of the fetus increases the risk of hypertension, coronary artery disease, and heart dysfunction [17, 20–25]. Animal models of gestational hypoxia [26–28] have been generated to investigate the underlying mechanisms of cardiovascular programming of the offspring. Intrauterine hypoxia is one of the most important obstetric complications that reduce fetal growth, which increases the risk of cardiovascular disease in the adult [20–25]. Animal models have shown that gestational hypoxia increases the risk of ischemia/reperfusion injury of adult rat hearts [26, 29–33], hypertension and cardiac dysfunction [34] and altered cardiomyocyte endowment of adult guinea pigs [35], and altered systemic vascular resistance in adult rats [33]. Thus, the impact of intrauterine stress such as prenatal hypoxia increases the vulnerability of the offspring to a lifetime of cardiovascular disease [20, 21].

Mitochondria are highly susceptible to hypoxia, which leads to respiratory chain dysfunction [36, 37]. We previously reported a decrease in cardiac CIV mRNA/protein expression and CIV activity of left ventricles along with a decrease in oxygen consumption rates of freshly isolated cardiac cells from hearts of male offspring guinea pigs following exposure to prenatal hypoxia [38]. This study demonstrated that prenatal hypoxia decreases cardiac performance of adult offspring guinea pigs, which was also accompanied by decreased cardiac CIV activity and respiratory function [34].

The goal of the current study was to more fully characterize the programming effects of prenatal hypoxia on cardiac mitochondria of adult guinea pig hearts in an animal model that exhibits sex-related differences in contractile dysfunction in the adult offspring [34]. Mitochondrial indices such as mitochondrial DNA content, CI-V protein expression, and CI and CIV activity were measured in both the left ventricle of the heart and in cardiomyocytes isolated from hearts of male and female adult guinea pigs. We propose that mitochondrial programming of cardiomyocytes of fetal hearts is initiated *in utero* by prenatal hypoxia as an underlying cause of mitochondrial and contractile dysfunction in the adult.

## 2. Methods

All animal procedures were approved by the University of Maryland Institutional Animal Care and Use Committee in accordance with the Association for Assessment and Accreditation of Laboratory Animal Care—accredited procedures (Animal Welfare Assurance No. A3200-01).

### 2.1. Animal Model

Pregnant guinea pigs were generated by mating multiple females with one male following the presence of an open vaginal membrane. Females were kept with males for a maximum of 48 hours or until the presence of vaginal membrane closure. Gestational age was estimated by palpation [39] and then confirmed at the time of delivery. Pregnant guinea pigs were exposed to either normoxia (room air, 21% O_2_) for the entire gestation or hypoxia (HPX, 10.5% O_2_, duration of 14 d) at 50 d gestation until delivery (term = 65 d). Pups were vaginally delivered and removed from the HPX chamber upon birth, and both male and female offspring were raised in a NMX environment. Animals were weighed at birth and weaned at 30 d old, and body weight and food and water intake rates were measured in 3 d intervals until 90 d old, when tissues were obtained. To remove the heart, guinea pig offspring were anesthetized with ketamine (80 mg/kg, s.c.) and xylazine (10 mg/kg, s.c.), and a thoracotomy was performed following an abdominal skin injection of lidocaine (1%). Hearts were excised and weighed and either dissected into left and right ventricles and frozen in liquid N_2_ or mounted onto a perfusion apparatus for collection of cardiomyocytes.

### 2.2. Cardiomyocyte Isolation

To obtain cardiac cells, hearts were excised from male and female offspring, immediately placed in iced physiological buffer solution (PBS), and mounted via the aorta onto a glass cannula of a Langendorff heart perfusion apparatus [34]. Using a modified procedure for isolating fetal sheep cardiac cells [40], hearts were retrograde-perfused at 37°C with a low Ca^2+^ (no Ca^2+^ added) Tyrode's solution (composition (in mM): 140 NaCl, 5 KCl, 10 HEPES, 10 glucose, and 1 MgCl_2_, pH 7.35) without enzymes for 5 minutes, followed by Tyrode's solution containing enzymes (collagenase (80 U/ml), protease (0.59 U/ml), and albumin (1 mg/ml)) for 12 min. This was followed by perfusion with Kraft-Bruhe (KB) buffer (composition (mM): 30 KCl, 10 HEPES, 10 glucose, 74 potassium glutamate, 20 taurine, 1.5 MgSO_4_·7H_2_O, 0.5 EGTA, and 30 KH_2_PO4, pH 7.37) for 5 minutes to wash out the enzymes. Hearts were removed from the apparatus and placed in a beaker containing warmed KB buffer for gentle mincing to release cells from the heart. Cells were filtered through a 150 *μ*m nylon mesh and transferred to a 15 ml centrifuge tube. Cells then were washed two times with KB buffer by centrifugation (200-250 g, 10 minutes), resuspended in 5 ml DMEM (Life Technologies, #12320-032), and pelleted by centrifugation at 1,200 g for 10 minutes. The final cell pellet was frozen in liquid N_2_ until assayed.

### 2.3. Mitochondrial Isolation

Mitochondrial proteins of left ventricular heart tissue and of cardiomyocytes were obtained from separate groups of 90 d old prenatally NMX or HPX guinea pigs. For assays of CI-V Western immunoblotting and CI and CIV activity assays, the mitochondrial fraction was isolated using a standard differential centrifugation protocol [41, 42]. Briefly, the frozen heart tissues (20-30 mg) were ground to a fine powder in liquid N_2_, and the frozen cardiomyocytes from separate animals were washed with 9 volumes of ice-cold PBS. Both were then separately resuspended in 1 ml of ice-cold Homogenization Buffer (0.25 M sucrose, 5 mM HEPES, and 1 mM EDTA, pH 7.2) and homogenized for 10 minutes at 4°C. Samples were centrifuged twice at 600 g for 10 minutes at 4°C to remove cellular debris. The supernatant was recentrifuged at 12,500 g for 10 min to generate an enriched mitochondrial fraction. The pellet containing the mitochondrial fraction was resuspended in 1x RIPA Lysis buffer supplemented with a protease inhibitor (Bio-Rad, Hercules, CA) for Western Blot or was solubilized with 0.1 mM N-Dodecyl *β*-D-maltoside (Sigma-Aldrich, St. Louis, MO) for complex activity assays. Total protein concentration of each sample was determined by the Bio-Rad Protein Assay (Bio-Rad).

### 2.4. Quantitative Real-Time PCR (qRT-PCR) of Mitochondrial DNA

Mitochondrial DNA (mtDNA) content was measured as an index of mitochondrial density. Total genomic DNA was isolated using the QIAamp DNA Mini Kit (Qiagen, Hilden, Germany) according to the manufacturer's instructions from left ventricular heart tissue prenatally exposed to NMX or HPX of 90 d old guinea pigs. DNA concentration was determined by NanoDrop (Thermo Fisher Scientific, Waltham, MA). Relative quantification of mtDNA content for each group was determined by qRT-PCR using primers for a mitochondrial gene (mt-ND1, forward 5 ′- CTAAAAACCCTTGCGCTCAC -3 ′; reverse 5 ′-TGGGAAGGGAAATGTGTCAT -3 ′) and a nuclear gene (*β*-actin, forward 5 ′-ACTCTCCACCTTCCAGCAGA -3 ′; reverse 5 ′-AAAGCCATGCCAATCTCATC-3 ′). qPCR was performed with a two-step cycling program by using the SYBR Green ROX™ qPCR Mastermix (Qiagen) and read on the QuantStudio 3 Real-time PCR System (Thermo Fisher). Gene expression was quantified by using the 2^-ΔΔCt^ method [43].

### 2.5. Blue Native Gel Electrophoresis and Western Blot Analysis

#### 2.5.1. Blue Native Gel Electrophoresis

Blue Native one-dimensional electrophoresis was performed for identification of whole respiratory complexes in isolated mitochondrial fractions of left ventricles of NMX and HPX offspring hearts. Isolated mitochondrial membrane fractions were isolated as previously described. The pellet was solubilized with 0.1 mM N-Dodecyl *β*-D-maltoside (Sigma-Aldrich, St. Louis, MO) similar to that for activity assays. To separate the respiratory chain complexes, 30 *μ*g of mitochondrial protein was loaded onto a high-resolution Clear Native PAGE of 3-12% Bis-Tris gradient gel (Thermo Fisher Scientific). The gel was stained with Coomassie dye and destained according to standard methods. The bands of individual complexes were visualized by the ChemiDoc Touch Imaging System (Bio-Rad).

#### 2.5.2. Western Blot Analysis

Protein expression of mitochondrial respiratory Complexes I-V was measured by Western immunoblot. Mitochondrial protein fractions obtained from left ventricular heart tissues and cardiomyocytes from separate groups of prenatally NMX and HPX 90 d old offspring were run on separate gels. Mitochondrial proteins (4 *μ*g) were separated on 4-15% precast gradient gels (Bio-Rad) and then transferred to PVDF membranes. The membranes were blocked with 5% nonfat milk in TBST for 2 hrs, incubated overnight at 4°C with primary antibody diluted in 5% nonfat milk in TBST, and then detected using an appropriate peroxidase-conjugated secondary antibody. Protein bands were targeted with an antibody cocktail (1 : 500) containing antibodies for complex subunits (I: NDUFB8, 20 kDa MW; II: SDHB, 30 kDa MW; III: UQCRC2 48 kDa MW; IV: MITCO1, 40 kDa MW; and V: ATP5a, 55 kDa MW) (Abcam, Cambridge, MA) and polyclonal anti-VDAC (voltage-dependent anion channel) antibody (1 : 2,000, Boster Biological Technology Co., Pleasanton, CA) and visualized by the ChemiDoc Touch Imaging System (Bio-Rad). Band densities were quantified by the Bio-Rad Image Lab System and normalized to the loading control, VDAC, to confirm equal loading.

### 2.6. Complex I and IV Activity Assays

Complex I and IV enzyme activity rates were measured using mitochondrial protein fractions of left ventricular heart tissue and cardiomyocytes isolated from hearts of separate groups of 90 d old prenatally NMX or HPX guinea pigs. Complex I enzyme activity was measured as the oxidation of NADH to NAD+ with a Complex I Enzyme Activity Microplate Assay Kit from Abcam (Cambridge, MA), an assay that measures the diaphorase-type activity of Complex I, which is independent of ubiquinone and rotenone sensitivity. Briefly, mitochondrial proteins were isolated as previously described, and 5 *μ*g was added to the microplate wells precoated with Complex I-specific antibody. After 3 hrs of incubation at 25°C, substrate (NADH and dye) was added to the wells, and OD values measured at 450 nm were recorded. Enzyme activity was expressed as the change in OD values per minute per mg protein.

Complex IV (cytochrome c oxidase) activity is responsible for reduction of O_2_ to H_2_O and is a measure of the oxidative capacity of the respiratory chain [44]. Cytochrome c oxidase activity was measured colorimetrically by monitoring the rate of oxidation of reduced cytochrome c (ferrocytochrome c). Briefly, the mitochondrial protein amount (2-4 *μ*g) was optimized to generate a reaction rate that followed first order kinetics, which is related to chemical reactions that are dependent on the molar concentration of one reactant. Following optimization, mitochondrial proteins (3 *μ*g) were added to a 96-well plate containing the assay buffer (10 mM Tri-HCl, pH 7.0, and 120 mM KCl plus 0.04 mM reduced cytochrome c (Sigma-Aldrich, St. Louis, MO) following reduction by 3 mM dithiothreitol. The OD values generated by oxidation of the reduced cytochrome c were measured as a decrease in absorbance at 550 nm in a 96-well plate reader (BioTek, Winooski, VT) at 10 sec intervals. Characteristics of first order kinetics were observed as cytochrome c oxidase activity was inhibited by generation of the ferricytochrome c product from oxidation of the ferrocytochrome c, and the reaction was slowed. The reaction rates of each sample were directly determined from a tangent drawn on the reaction curve at the 3 min time interval. Thus, the observed kinetics of the reaction reflects the complex interaction of the effect of decreasing substrate concentration and increasing product inhibition on cytochrome c oxidase [45]. Cytochrome oxidase activity (units/mg protein) = ΔOD/time (Δ*t*)/*ε*∗protein (mg), (*ε* = 7.04 mM^−1^ cm^−1^).

## 3. Results

### 3.1. Animal Model

**Figure 1:**
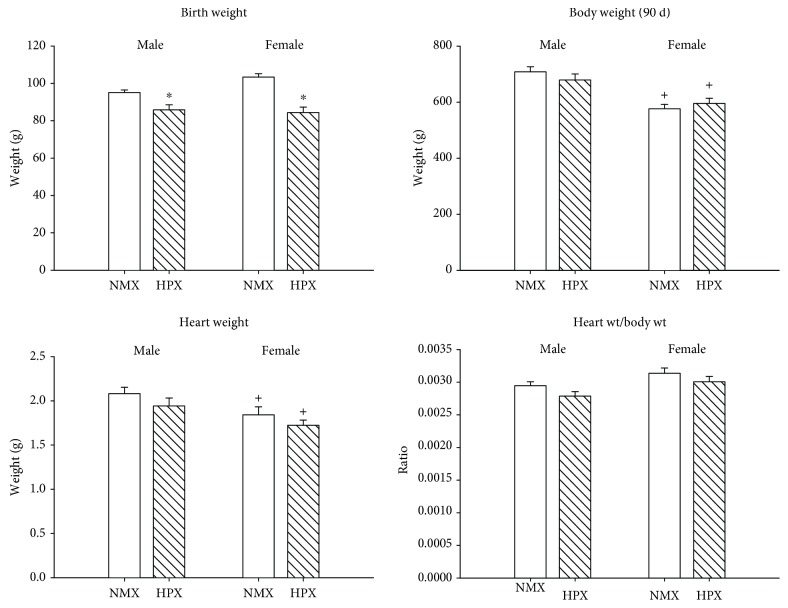
Effects of prenatal hypoxia (HPX) on body weight, heart weight, and relative heart weight (heart wt/body wt). Values were measured in male and female offspring exposed to normoxia (NMX, open bars) or prenatal HPX (10.5% O_2_, 14 d, hatched bars). ^∗^*P* < 0.05 indicates significant difference from the NMX control and ^+^*P* < 0.05 from male.

Neonates exposed to prenatal HPX exhibited a decrease in birth weight in both male (*N* values: 8 NMX, 8 HPX) and female (*N* values: 8 NMX, 8 HPX) offspring compared to age-matched NMX controls (Figure 1). At 90 d old, offspring body weights were no different between treatment groups for both sexes. There were no differences in food or water intake rates between NMX and prenatally HPX offspring as reported in a previous study [3]. There were no morphological differences in total heart weight, left ventricular weight, heart weight/body weight ratios (Figure 1), or left ventricular weight/heart weight ratios (male:  0.74 ± 0.01 vs. 0.76 ± 0.01; female: 0.76 ± 0.01 vs. 0.76 ± 0.01, NMX vs. HPX, respectively) between NMX and HPX animals. This is identical to the growth profile we previously reported [34], in which body weights of prenatally HPX offspring were reduced at birth and 30 days old but were similar at 60 and 90 days old. Sex differences in body weights were found at 90 days old, with males exhibiting greater weight than females.

### 3.2. Mitochondrial Content

**Figure 2:**
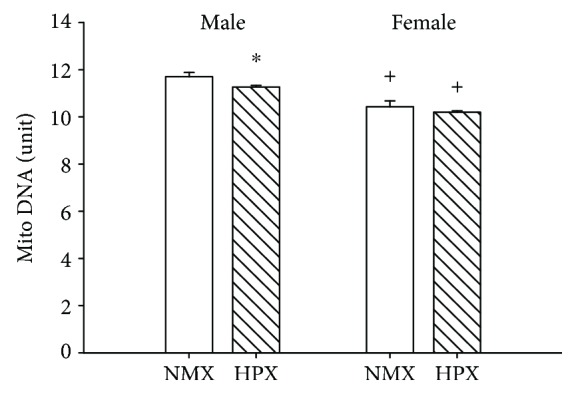
Effects of prenatal hypoxia (HPX) on mitochondrial (mito) DNA content. Cardiac mitochondria were obtained from left ventricular heart tissue from male and female offspring exposed to normoxia (NMX, open bars) or prenatal HPX (10.5% O_2_, 14 d, hatched bars). ^∗^*P* < 0.05 indicates significant difference from the NMX control and ^+^*P* < 0.05 vs. male.

Mitochondrial content was measured by qRT-PCR of mitochondrial DNA. Prenatal HPX significantly (*P* < 0.05) reduced mtDNA (Figure 2) in left ventricular heart tissue of male offspring. There was a significant decrease in mito DNA content in female compared to male left ventricles although prenatal HPX had no effect on mtDNA content in female heart tissue.

### 3.3. Complex I-V Subunit Protein Expression

**Figure 3:**
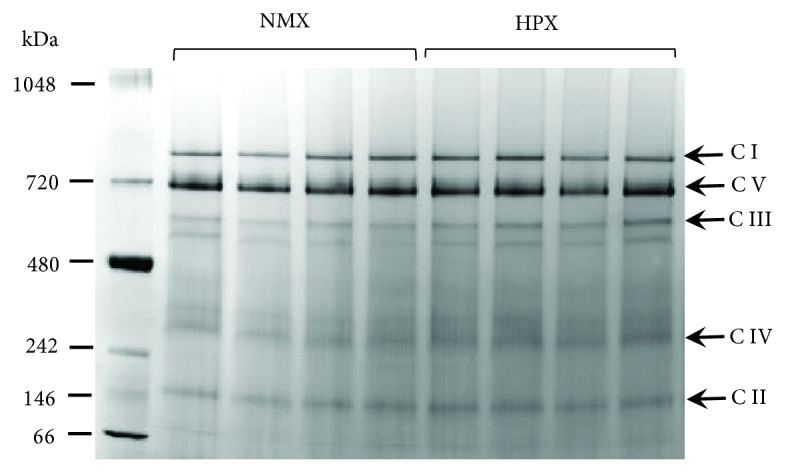
One-dimensional Blue Native-PAGE of mitochondrial fractions of offspring heart ventricles. Cardiac mitochondria were isolated from normoxic (NMX, lanes 2-5) or prenatally exposed hypoxic (HPX (10.5% O_2_, lanes 6-9) guinea pig offspring. Labels CI-V correspond to whole respiratory complexes extracted from cardiac mitochondria based on MW. Lane 1 is the MW marker.

**Figure 4:**
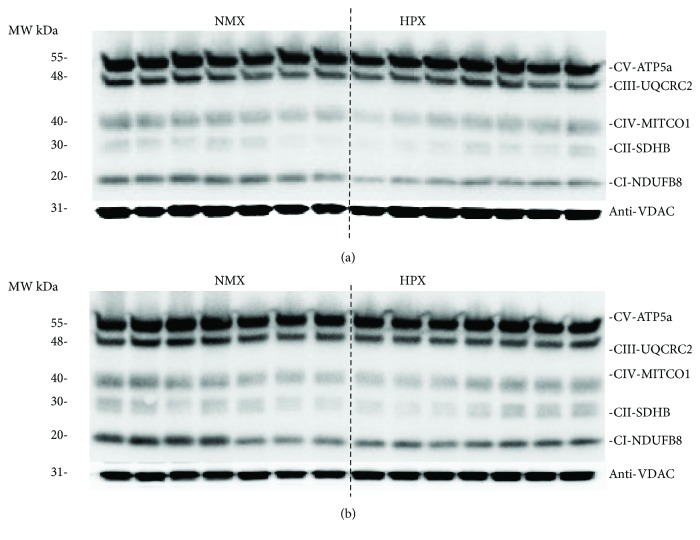
Effects of prenatal hypoxia (HPX) on cardiac mitochondrial complex (I-V) expression of left ventricular heart tissue from male (a) and female (b) offspring. Hearts were obtained from offspring exposed to normoxia (NMX, open bars) or prenatal HPX (10.5% O_2_, 14 d, hatched bars). Western immunoblots illustrate protein expression of representative subunits of Complexes I-V using an antibody cocktail.

**Figure 5:**
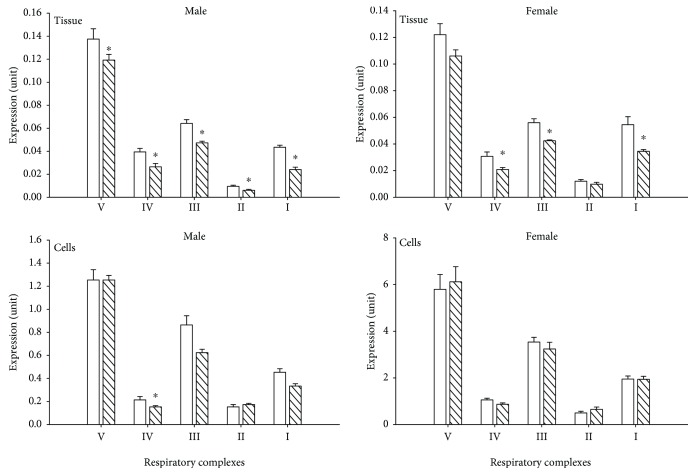
Western blot analysis of mitochondrial complex (I-V) subunit expression. Cardiac mitochondria were obtained from tissue and isolated cells of male and female offspring (normoxia (NMX), open bars; prenatal HPX (10.5% O_2_, hatched bars). Densities were normalized to VDAC (expression units). ^∗^*P* < 0.05 from the NMX control. Complex I-V subunits are NDUFB8, SDHB, UQCRC2, MITCO1, and ATP5a, respectively.

Figure 3 is a one-dimensional BN-PAGE gel illustrating the presence of fully assembled respiratory Complexes I-V extracted from mitochondrial fractions of left ventricular tissue homogenates. Each of the bands correspond to an individual intact respiratory complex and were confirmed by the MW. This indicates that all five complexes are fully assembled following extraction and exposure to hypoxia. The effect of HPX treatment on expression of individual complexes was investigated by performing Western blot analysis of individual subunits corresponding to Complexes I-V. Representative Western blots of Complex I-V subunit expression for left ventricles for NMX and HPX, male and female hearts, are illustrated in Figure 4.  Figure 5 illustrates the graphic analysis of the effects of HPX on representative mitochondrial CI-V subunit expression in both left ventricular homogenates and freshly isolated cells of offspring hearts (NMX: 7 males, 7 females; HPX: 7 males, 7 females). In tissue homogenates, prenatal HPX significantly inhibited (*P* < 0.05) the protein subunit expression of all 5 complexes in male offspring and subunits of CI, CIII, and CIV in female offspring (Figure 5). In cardiac cells derived from male hearts, prenatal HPX significantly inhibited (*P* < 0.05) CIV subunit expression only. In cells from female hearts, prenatal HPX had no significant effect on expression of any of the 5 complexes.

### 3.4. Complex (C) I and IV Activity Rates

**Figure 6:**
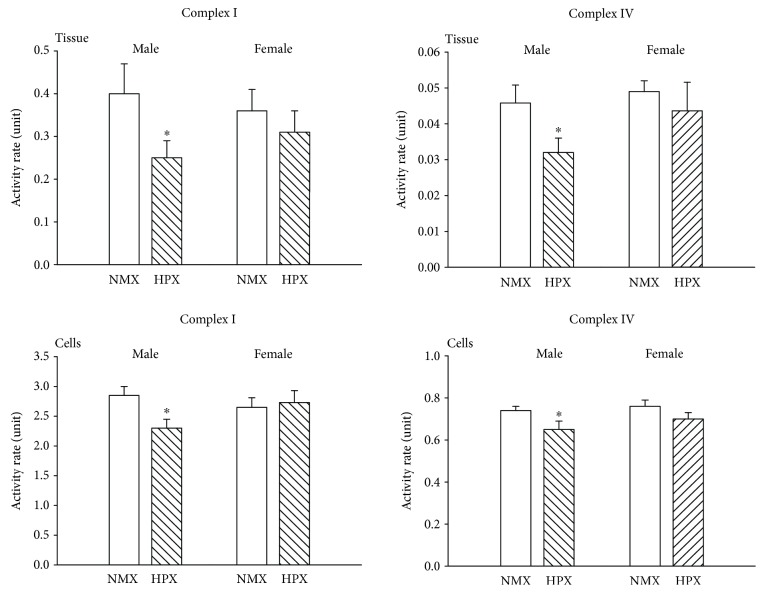
Effects of prenatal hypoxia (HPX) on Complex I and IV activity rates of cardiac mitochondria from left ventricular heart tissue and cardiomyocytes of hearts from male and female offspring exposed to normoxia (NMX, open bars) or prenatal HPX (10.5% O_2_, 14 d, hatched bars). The activity rate (unit) was expressed as the oxidation rate of substrate per min per mg mitochondrial protein (CI: oxidation of NADH; CIV: oxidation of ferrocytochrome c). ^∗^*P* < 0.05 indicates significant difference from the NMX control.

CI activity was significantly reduced in both left ventricular heart tissue (NMX: *N* = 16, 8 males, 8 females; HPX: *N* = 16, 8 males, 8 females) and cardiomyocytes (NMX: *N* = 16, 8 males, 8 females; HPX: *N* = 16, 8 males, 8 females) from male hearts of prenatally HPX offspring compared to their NMX controls (Figure 6). Similarly, CIV activity rates were reduced in both heart tissue and cardiomyocytes of male hearts of prenatally HPX offspring. In contrast, prenatal HPX had no effect on either CI or CIV activity in either female heart tissue or cardiomyocytes from female hearts.

## 4. Discussion

This study presents evidence that prenatal HPX induces alterations in cardiac mitochondria that are manifested in offspring hearts as decreased mitochondrial content, decreased CIV expression, and decreased CI/CIV activity. Secondly, there is a sexual dimorphism in response of the mitochondria to prenatal HPX favoring female preservation of complex expression and activity compared to males. While prenatal HPX reduces body weight in both sexes at birth, following catch-up growth, there are no differences in body weight, heart weight, or relative heart weight at 90 days old. Despite this, there were significant changes in indices of cardiac mitochondrial function.

### 4.1. Programming of Cardiac Mitochondrial Function

Efficient delivery of ATP via oxidative phosphorylation is critical for maintaining normal heart function [1]. The impact of prenatal HPX on cardiac mitochondria of offspring identifies a programming effect that may contribute to an underlying cause of heart dysfunction. The current study provides new information on indices of mitochondrial function that are altered by exposure to prenatal HPX and confirms our previous result [34]. We have previously reported that prenatal HPX programs contractile dysfunction in male but not female offspring hearts in association with a decrease in CIV activity [34, 38] and oxygen consumption of cardiac cells [34].

A decrease in mitochondrial DNA content was measured in prenatally HPX males but not females. This may reflect an effect of prenatal HPX on mitophagy since HPX is an important regulator of mitochondrial degradation [46]. Under conditions of chronic HPX, the fetal heart may regulate its mitochondrial content by removing defective mitochondria as a cardioprotective mechanism [46]. It is unknown whether the sustained decrease in mitochondrial content of prenatally HPX offspring hearts is due to changes occurring in the fetal heart and sustained postnatally or occurring at the time of birth in response to the hyperoxic environment into which the fetus is born. Regardless, it suggests that prenatal HPX contributes to the sustained changes in the offspring.

Prenatal HPX decreased subunit expression of CI-CV in male and CI, CIII, and CIV in female heart tissue homogenates but only CIV in freshly isolated cardiomyocytes of male hearts. Cells were isolated from whole heart tissue to identify the cell-specific effects of prenatal HPX. It should be noted that the intact complex is made up of several subunits, whose numbers vary depending on the complex. Altered subunit expression may impact assembly of the respiratory complex and its functional activity [47]. The small differences in subunit protein expression with HPX may indicate that the reduced protein expression is of small physiological significance. However, differences in transcriptional regulation of complex subunits may manifest in several other aspects of mitochondrial function such as CI/CIV activity measured in the current study and a decreased oxygen consumption rate of intact cells, as reported in our previous study [38]. The difference of prenatal HPX on complex subunit expression between whole tissue and isolated cells may reflect the difference in heterogeneous versus homogeneous cell populations. In isolated cardiac cells, the decrease in CIV subunit expression is small but significant in males but not females, consistent with the sex differences in CIV activity. However, CI activity was reduced but in the absence of decreased CI subunit expression, suggesting posttranslational regulation of enzyme activity. The decrease in both CI and CIV activities may also reflect several aspects of mitochondrial function besides altering energy supply [48]. For example, decreased CI activity can increase the premature leakage of electrons from the electron transport chain to generate superoxide anions, resulting in mitochondrial oxidative stress [37, 49–51]. Additionally, this may increase the NADH/NAD+ ratio due to decreased NADH consumption by CI, with the resulting increase in NADH inhibition to enzymes in the TCA cycle [52, 53]. Data from the current study corroborate our previous report that both the maximal oxygen consumption rate and respiratory reserve capacity were reduced in cells derived from prenatally HPX male but not female hearts [34]. Thus, prenatal HPX may change the intrinsic properties of the respiratory complexes in the offspring heart and thereby decrease respiratory function.

### 4.2. Implications of Programming on Heart Function in the Offspring

The mechanism of programming of mitochondrial dysfunction in offspring hearts is poorly understood because of the maturational changes the fetal heart undergoes as well as the environmental changes to which the neonate is exposed. The mitochondria of the fetal heart undergo a maturational process that involves both structural organization and functional changes in preparation of the metabolic switch that occurs at birth [5, 54]. Intrauterine HPX can disrupt this process directly, by generating mitochondria-derived ROS [37, 49–51], or indirectly, by disrupting transcriptional regulation of mitochondrial proteins [54] and/or altering complex activity [55, 56]. It is unclear whether HPX conditions disrupt mitochondrial respiration via enhanced ROS generation, causing oxidative damage, or via altered electron flux as a result of reduced complex activity. Regardless, there is a close association between altered cardiac mitochondrial and contractile function in the hearts of offspring [34], which suggests a programming effect of prenatal HPX as an underlying mechanism.

### 4.3. Sex Differences in Mitochondrial Function

While mitochondrial sex differences in response to prenatal HPX were present, the mechanisms underlying these differences are unclear. Sex differences in response to prenatal HPX may have their origins in the fetus, offspring, or both. Sex differences of the fetus are associated with both the genetic makeup of the embryo/fetus [57, 58] and underlying differences in fetal hormone levels such as testosterone and estrogen [59–61]. Thus, differences in response to HPX in the offspring may originate from either the genetic background or the differences in fetal hormones, which may contribute to the differences in epigenetic mechanisms induced by HPX [57]. Further, the placenta may contribute to the sexual dimorphism in its response to intrauterine stress [57] due to differences in hormone synthesis (i.e., cortisol and testosterone) [62]. We have previously shown that HPX reduces CI and CIV activities in male versus female placentas under identical conditions as the current study [63]. Thus, differences in placental hormones may contribute to the fetal response to intrauterine hypoxia and its subsequent effects in the offspring.

Differences in sex steroid hormones in the reproductively mature adult may also contribute to the sex differences in mitochondrial complex activity. The mitochondria are considered major targets of cardioprotective signaling by estrogen, whose levels differ between male and female. It is reasonable to consider that a secondary effect of postpubertal steroid levels in female offspring may protect against and even reverse the altered mitochondrial effects programmed by prenatal HPX. Several studies have demonstrated estrogen's protective effects by enhancing the antioxidant capacity in female compared to male mitochondria [60, 61, 64, 65] as well as stimulating mitochondrial biogenesis by upregulating PGC-1*β* and NRF1 via nuclear gene transcription by binding to ER*α*/*β* (61).

In conclusion, mitochondrial dysfunction is a contributing factor to heart dysfunction and eventual heart failure as a result of inadequate energy delivery to the myofibrils [1]. Sex differences in mitochondrial protein expression and activity in response to HPX may contribute to sex difference in heart failure [66] and cardiovascular disease [67] with age-matched men having a higher incidence than women. Extensive study has now identified multiple mechanisms by which intrauterine HPX can program heart dysfunction in the adult [27, 28, 68]. We propose that the cardiac mitochondria are critical downstream target organelles whose intrinsic properties of the respiratory chain complexes are altered by intrauterine HPX. Thus, the programming of the cardiac mitochondria by prenatal HPX may play a central role in cardiac dysfunction in the adult, given its importance in cell metabolism, energy supply, and contractile function. Further, the dysregulation of cardiac mitochondrial function, initiated *in utero*, may contribute to the vulnerability of the offspring to factors contributing to cardiovascular disease [66, 67] and heart failure [16, 66].

## Data Availability

The data used to support the findings of this study are available from the corresponding author upon request.
